# Inference and quantile regression for the unit-exponentiated Lomax distribution

**DOI:** 10.1371/journal.pone.0288635

**Published:** 2023-07-18

**Authors:** Aisha Fayomi, Amal S. Hassan, Ehab M. Almetwally

**Affiliations:** 1 Faculty of Science, Department of Statistics, King Abdulaziz University, Jeddah, Saudi Arabia; 2 Faculty of Graduate Studies for Statistical Research, Cairo University, Giza, Egypt; 3 Faculty of Business Administration, Delta University for Science and Technology, Gamasa, Egypt; 4 The Scientific Association for Studies and Applied Research, Al Manzalah, Egypt; University of Bradford, UNITED KINGDOM

## Abstract

In probability theory and statistics, it is customary to employ unit distributions to explain practical variables having values between zero and one. This study suggests a brand-new distribution for modelling data on the unit interval called the unit-exponentiated Lomax (UEL) distribution. The statistical aspects of the UEL distribution are shown. The parameters corresponding to the proposed distribution are estimated using widely recognized estimation techniques, such as Bayesian, maximum product of spacing, and maximum likelihood. The effectiveness of the various estimators is assessed through a simulated scenario. Using mock jurors and food spending data sets, the UEL regression model is demonstrated as an alternative to unit-Weibull regression, beta regression, and the original linear regression models. Using Covid-19 data, the novel model outperforms certain other unit distributions according to different comparison criteria.

## 1 Introduction

Lomax [1] established the Lomax (L) or Pareto II distribution to model data on business failure. This distribution has found widespread use in a variety of domains, including income and wealth disparity [2, 3], city size [4], the size distribution of computer files on servers [5], internet traffic [6], and receiver operating characteristic curve analysis [7]. Reference [8] claimed that the Lomax distribution is a good fit for modeling reliability issues because many of its characteristics can be understood in that context and could serve as an alternative to the well-known distributions used in reliability. Reference [9] utilized this distribution to model size spectrum data in aquatic ecology. Many descriptions are provided for the characterization of the Lomax distribution. It is referred to as a particular model of the Pearson Type VI distribution. It has also been thought of as a combination of the exponential and gamma distributions. The L model emerges as a limiting distribution of residual lifetimes at very old ages [10]. The L belongs to the family of decreasing failure rates in the context of lifetimes [11]. Reference [12] mentioned that the L distribution is a heavy-tailed alternative to the exponential, Weibull, and gamma distributions. Moreover, it belongs to the Burr family of distributions [13].

The cumulative distribution function (CDF) of the Lomax distribution is defined by:

$$\begin{array}{c} G\left(x;\lambda ,\delta \right)=1-{\left(1+\lambda x\right)}^{-\delta },\;\;\;x>0,\;\;\; \end{array}$$ (1)

where, λ, *δ* ∈ R^+^, are the scale and shape parameters respectively. The probability density function (PDF) of the Lomax distribution is as follows:

$$\begin{array}{c} g\left(x;\lambda ,\delta \right)=\lambda \delta {\left(1+\lambda x\right)}^{-(\delta +1)},\;\;\;x>0.\;\;\; \end{array}$$ (2)

From PDF (2), it is clear that the L distribution simplifies to:

1. The beta prime (or inverted beta) distribution for λ = 1, *δ* ≠ 1.
2. The log-logistic distribution for *δ* = 1, λ ≠ 1.
3. F(2,2) distribution for *δ* = λ = 1.

To gain a better match for data analysis in different disciplines, modified and expanded variants of the L distribution were established. The next are a few of these broad generalizations that are highlighted; expoentiated L [14], Marshall Olkin L distribution [15], McDonald L distribution [16], Weibull L distribution [17], gamma L distribution [18], exponentiated Weibull L distribution [19], Maxwell L distribution [20], and Nadarajah-Haghighi L distribution [21].

The interest in the present study with the exponentiated L (EL) distribution with an extra shape parameter *ϑ* compared to (1). The EL distribution has the following CDF and PDF:

$$\begin{array}{c} G\left(x;\Theta \right)={\left[1-{\left(1+\lambda x\right)}^{-\delta }\right]}^{\vartheta },\;\;\;x>0, \end{array}$$ (3)

$$\begin{array}{c} g\left(x;\Theta \right)=\delta \lambda \vartheta {\left(1+\lambda x\right)}^{-\delta -1}{\left[1-{\left(1+\lambda x\right)}^{-\delta }\right]}^{\vartheta -1},\;\;\;x>0, \end{array}$$ (4)

where Θ ≡ (*δ*, λ, *ϑ*) ∈ R^+^. The EL model provides well-known distributions for certain specific values of parameters. It includes Lomax distribution for *ϑ* = 1, and exponentiated Pareto distribution for λ = 1.

Recently, the development of new flexible probability distributions to provide well-fitting models to datasets with values ranging from 0 to 1, has piqued statisticians’ curiosity. These bounded distributions are necessary for modeling proportions, percentages, and probabilities. In applied disciplines, there is a strong need for the analysis of datasets on the (0, 1) for semi-parametric or parametric and regression models. the analysis of datasets on the (0, 1) for parametric, semi-parametric, and regression models is also in high demand. Furthermore, unit distributions allow extra flexibility throughout the unit interval without introducing new parameters to the fundamental distribution. The following are some of the most important unit distributions with diverse numbers of parameters: log-Lindley distribution [22], unit-Birnbaum-Saunders distribution [23], unit-Gompertz distribution [24], unit-inverse Gaussian distribution [25], unit-Weibull distribution [26], unit-Burr-XII distribution [27], unit-Gamma/Gompertz distribution [28], unit exponentiated half logistic distribution [29], unit power Burr X distribution [30] and unit inverse exponentiated Weibull distribution [31].

In this study, in light of the above, an inverse-exponential transformation is used to generate a new unit-flexible probability distribution with a three-parameter based on the EL distribution. The new distribution, named the unit exponentiated Lomax (UEL) distribution, may be utilized to evaluate a wide range of datasets having a value from zero to one. A quantile regression model is developed based on the parametrization of the UEL distribution in terms of the *μ*^*th*^ quantile. In light of the following facts, the UEL distribution is introduced:

1. To provide a novel distribution defined on (0,1) as a competitor to the existing bounded distributions;
2. The new distribution can take different hazard rate shapes, such as constant, increasing, decreasing, and bathtub;
3. It may be considered a model that is appropriate for fitting skewed data that may not be well fitted by other common distributions and can be used to address a wide range of issues in many fields;
4. To investigate key statistical features of the UEL distribution, such as entropy measures, probability-weighted moments (PWMs), moments, stress-strength (S-S) reliability, and incomplete moments (IM);
5. To investigate inferential features of the UEL distribution parameters using widespread estimation methodologies, such as maximum likelihood (ML), a maximum product of spacing (MPS), and Bayesian;
6. To examine the performance of the parameters using a simulation methodology;
7. Three real-data applications are examined: the first and second data sets are concerned with quantile regression modeling, while the third data set is concerned with data modeling.

The following is a description of the paper’s structure. Section 2 defines the suggested distribution. Section 3 describes its essential distributional features. The methodologies for estimating unknown parameters using various estimation approaches are covered in Section 4. In Section 5, a simulation analysis is conducted to assess the parameter estimates. The new quantile regression model based on the newly specified distribution is described in Section 6. Two real-world applications employing the suggested quantile regression model alongside additional famous regression models are shown in Section 7. The application of the UEL distribution to Covid-19 data in Section 7 reveals that it is preferred to the other seven unit distributions. In Section 8, the paper comes to a close.

## 2 Model description

In this section, a restriction of the EL distribution in the unit interval is done to introduce a new bounded distribution with support on (0, 1) referring to the UEL distribution. Several extension\ modifications have been done using some transformation, in the present work, a similar exponential transformation is used as provided in References [24–31].

Suppose that *Y* = *e*^−*X*^, where *X* is the EL distribution, then the CDF of the UEL distribution is determined as follows:

$$\begin{array}{c} \begin{array}{cc} F(y;\Theta ) & =P(Y\leq y)\\ & =P(e^{-X}\leq y)\\ & =P(-X\leq \text{ln}(y))\\ & =1-P(X\leq -\text{ln}(y))\\ & =1-F_{X}\left(-\text{ln}\left(y\right)\right)\\ & =\;\;1-{\left\{1-{\left(1-\lambda \;\text{ln}(y)\right)}^{-\delta }\right\}}^{\vartheta }. \end{array} \end{array}$$

Based on the previous equation, the CDF and PDF of the UEL distribution are provided in the following definition.

*Definition: A random variable Y is said to follow the UEL distribution with a set of parameters* Θ ≡ (*δ*, λ, *ϑ*) ∈ R^+^, *if it’s CDF and PDF, are given by*:

$$\begin{array}{c} F\left(y;\Theta \right)=1-{\left\{1-{\left(1-\lambda \;\text{ln}(y)\right)}^{-\delta }\right\}}^{\vartheta },\;\;\;\;\;\;\;\;\;0\;<y<1, \end{array}$$ (5)

and

$$\begin{array}{c} f\left(y;\Theta \right)=\frac{\lambda \delta \vartheta }{y}{\left(1-\lambda \;\text{ln}(y)\right)}^{-\delta -1}{\left\{1-{\left(1-\lambda \;\text{ln}(y)\right)}^{-\delta }\right\}}^{\vartheta -1},\;\;\;\;\;0\;<y<1. \end{array}$$ (6)

Note that, *F*(*y*; Θ) = 0, for *y* ≤ 0, and *F*(*y*; Θ) = 1, for *y* > 1. The survival function and hazard rate function (HRF) are as follows:

$$\bar{F}\left(y;\Theta \right)={\left\{1-{\left(1-\lambda \;\text{ln}(y)\right)}^{-\delta }\right\}}^{\vartheta };\;\;\;\;\;\;\;\;\;\;\;\;\;\;0\;<y<1,$$

$$h\left(y;\Theta \right)=\frac{\lambda \delta \vartheta }{y}{\left(1-\lambda \;\text{ln}(y)\right)}^{-\delta -1}{\left\{1-{\left(1-\lambda \;\text{ln}(y)\right)}^{-\delta }\right\}}^{-1}.$$

**Fig 1:**
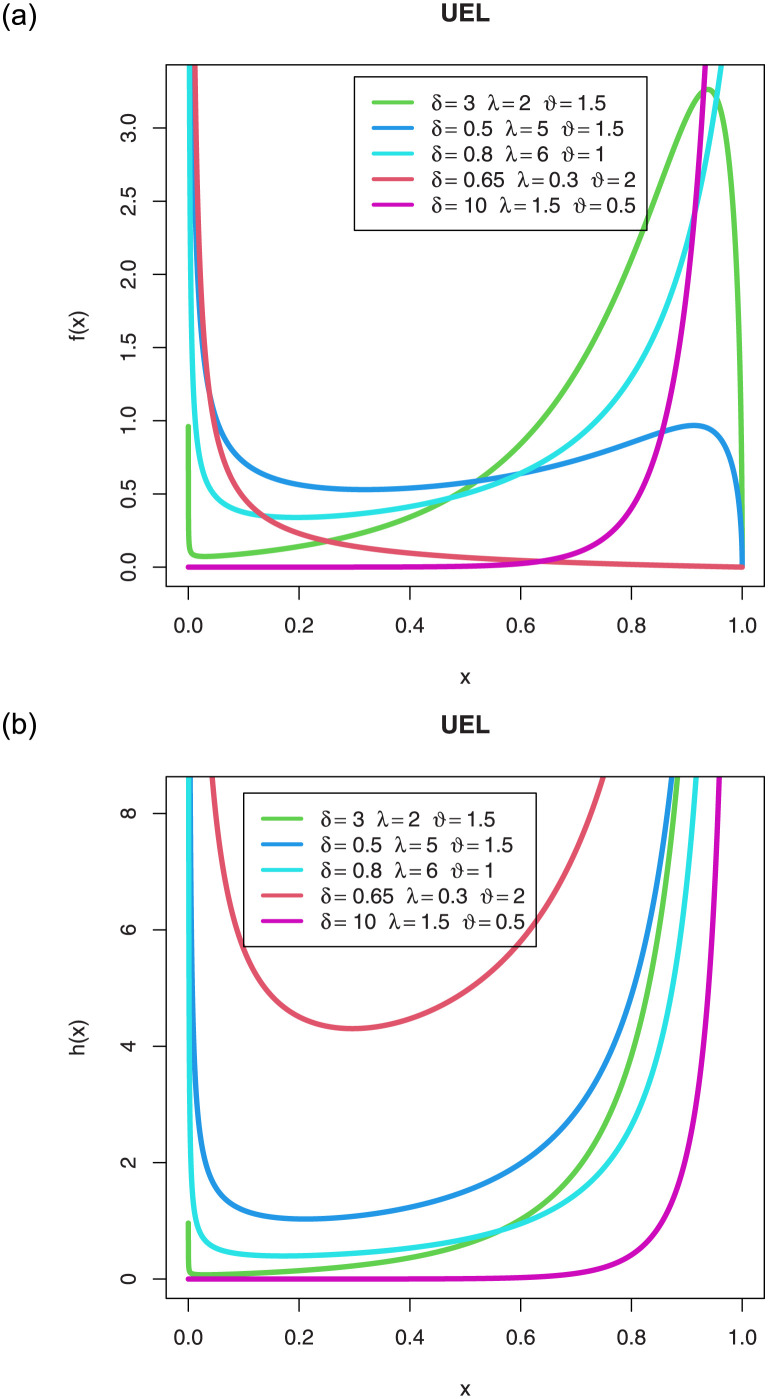
Density and HRF plots with different parameter values.

The density and HRF plots of the UEL distribution are given in Fig 1 for specific values of parameters.

From Fig 1, it can be concluded that the UEL density takes a variety of forms, such as left-skewed, U-shaped, unimodal, and J-shaped. Also, the HRF can be increasing, decreasing, J-shaped, or bathtub-shaped.

## 3 Characteristics of the UEL distribution

Specific of the UEL distribution’s structural features, such as PWM, moments and IM, quantile function, some entropy measures, and S-S reliability, are investigated in this section.

### 3.1 Probability weighted moments

The PWM is typically thought to be preferable to ordinary moments. The PWM is less sensitive to extreme values. When ML estimators are difficult to obtain, they are occasionally utilized. The class of PWM, denoted by Ψ_*h*,*s*_, for a random variable *Y*, is characterized as follows:

$$\begin{array}{c} \Psi_{h,s}=E\left[Y^{h}F{\left(y\right)}^{s}\right]=\int_{-\infty }^{\infty }y^{h}{\left(F\left(y\right)\right)}^{s}f\left(y\right)dy.\; \end{array}$$ (7)

Substituting (5) and (6) in (7), then the PWM of the UEL distribution is

$$\begin{array}{c} \;\Psi_{h,s}=\lambda \delta \vartheta \int_{0}^{1}y^{h-1}{\left(1-\lambda \;\text{ln}\left(y\right)\right)}^{-\delta -1}{\left\{1-{\left(1-\lambda \;\text{ln}\left(y\right)\right)}^{-\delta }\right\}}^{\vartheta -1}{\left[1-{\left\{1-{\left(1-\lambda \;\text{ln}\left(y\right)\right)}^{-\delta }\right\}}^{\vartheta }\right]}^{s}dy. \end{array}$$

Using the binomial expansion, where *s* is a positive integer, then Ψ_*h*,*s*_, is as follows:

$$\begin{array}{c} \Psi_{h,s}=\sum_{u=0}^{s}{\left(-1\right)}^{u}\left(\begin{array}{c} s\\ u \end{array}\right)\;\lambda \delta \vartheta \int_{0}^{1}y^{h-1}{\left(1-\lambda \;\text{ln}\left(y\right)\right)}^{-\delta -1}{\left\{1-{\left(1-\lambda \;\text{ln}\left(y\right)\right)}^{-\delta }\right\}}^{\vartheta \left(u+1\right)-1}dy.\;\; \end{array}$$ (8)

Using the following binomial expansion in the last term of (8)

$$\begin{array}{c} {\left\{1-{\left(1-\lambda \;\text{ln}\left(y\right)\right)}^{-\delta }\right\}}^{\vartheta \left(u+1\right)-1}=\sum_{m=0}^{\infty }\left(\begin{array}{c} \vartheta \left(u+1\right)-1\\ m \end{array}\right)\;{\left(-1\right)}^{m}{\left(1-\lambda \;\text{ln}\left(y\right)\right)}^{-\delta m}. \end{array}$$ (9)

Inserting (9) in (8) leads to

$$\Psi_{h,s}=\sum_{u=0}^{s}\sum_{m=0}^{\infty }{\left(-1\right)}^{u+m}\left(\begin{array}{c} s\\ u \end{array}\right)\left(\begin{array}{c} \vartheta \left(u+1\right)-1\\ m \end{array}\right)\;\lambda \delta \vartheta \int_{0}^{1}y^{h-1}{\left(1-\lambda \;\text{ln}\left(y\right)\right)}^{-\delta \left(m+1\right)-1}dy.$$

Let *z* = −λ ln(*y*), then the UEL distribution’s PWM is:

$$\;\Psi_{h,s}=\sum_{u=0}^{s}\sum_{m=0}^{\infty }{\left(-1\right)}^{u+m}\left(\begin{array}{c} s\\ u \end{array}\right)\left(\begin{array}{c} \vartheta \left(u+1\right)-1\\ m \end{array}\right)\;\lambda \delta \vartheta \int_{0}^{\infty }e^{\frac{-hz}{\lambda }}{\left(1+z\right)}^{-\delta \left(m+1\right)-1}dz.$$

Using exponential expansion, in the previous equation, then the PWM of the UEL distribution becomes

$$\Psi_{h,s}=\sum_{u=0}^{s}\sum_{m,j=0}^{\infty }\frac{{\left(-1\right)}^{u+m+j}h^{j}\delta \vartheta }{\lambda^{j}\;j!}\left(\begin{array}{c} s\\ u \end{array}\right)\left(\begin{array}{c} \vartheta \left(u+1\right)-1\\ m \end{array}\right)\;B\left(1+j,\delta \left(m+1\right)-j\right),$$

where B(.,.) stands for beta function (BF).

### 3.2 Moments & associated measures

If *Y* has the PDF (6), then the *s*^*th*^ moment is derived as

$$\begin{array}{c} \mu_{s}^{\prime }=\lambda \delta \vartheta \int_{0}^{1}y^{s-1}{\left(1-\lambda \;\text{ln}\left(y\right)\right)}^{-\delta -1}{\left\{1-{\left(1-\lambda \;\text{ln}\left(y\right)\right)}^{-\delta }\right\}}^{\vartheta -1}dy.\; \end{array}$$ (10)

Let *z* = −λ ln(*y*), and use the exponential expansion, then the previous equation is

$$\mu_{s}^{\prime }=\sum_{k,j=0}^{\infty }\frac{{\left(-1\right)}^{k+j}{\left(s\right)}^{j}\delta \vartheta }{j!\lambda^{j}}\left(\begin{array}{c} \vartheta -1\\ k \end{array}\right)\;B[1+j,\delta \left(k+1\right)-j].$$

The *s*^*th*^ central moment, say *μ*_*s*_, of a given random variable *Y*, is defined by:

$$\mu_{s}=E{\left(Y-\mu_{1}^{\prime }\right)}^{s}=\sum_{i=0}^{s}{\left(-1\right)}^{i}\left(\begin{array}{c} s\\ i \end{array}\right){\left(\mu_{1}^{\prime }\right)}^{i}\mu_{s-i}^{\prime }.$$

Numerical values for certain parameter values, (a)(λ = 0.5, *ϑ* = 0.5, *δ* = 1.5), (b)(λ = 0.5, *ϑ* = 1.5, *δ* = 1.5), (c)(λ = 0.5, *ϑ* = 1.5, *δ* = 0.5), (d)(λ = 2, *ϑ* = 0.5, *δ* = 1.5), (e)(λ = 2, *ϑ* = 1.5, *δ* = 1.5), (f)(λ = 2, *ϑ* = 1.5, *δ* = 0.5), of the first four moments, variance (*σ*^2^), coefficient of skewness (CS) and coefficient of kurtosis (CK) of the UEL distribution are listed in Table 1.

**Table 1:** Some moments of the UEL distribution.

Measures	(a)	(b)	(c)	(d)	(e)	(f)
$\mu_{1}^{\prime }$	0.172	0.169	0.024	0.376	0.527	0.124
$\mu_{2}^{\prime }$	0.095	0.07	0.0076	0.271	0.328	0.059
$\mu_{3}^{\prime }$	0.063	0.037	0.0036	0.211	0.228	0.035
$\mu_{4}^{\prime }$	0.046	0.023	0.0021	0.172	0.169	0.024
*σ* ^2^	0.065	0.041	0.0071	0.129	0.051	0.044
CS	1.448	1.399	5.225	0.254	0.158	1.857
CK	3.928	4.239	34.814	1.448	1.344	5.573

From Table 1, it can be concluded that the moment values decrease with increasing value of *ϑ*, for fixed values of λ, *δ*. Also, according to the values of CS and CK, the distribution is right-skewed, platykurtic, and leptokurtic.

### 3.3 Quantile function

For *q* ∈ (0, 1), the quantile function (QF) of *Y* is obtained by inverting (5) as follows:

$$q=1-{\left\{1-{\left(1-\lambda \;\text{ln}\left(y_{q}\right)\right)}^{-\delta }\right\}}^{\vartheta },$$

which provides;

$$\begin{array}{c} y_{q}=\text{exp}\;[\frac{1}{\lambda }\left\{1-{\left(1-{\left(1-q\right)}^{\frac{1}{\vartheta }}\right)}^{-1/\delta }\right\}],\;0<q<1. \end{array}$$ (11)

The first quantile, median and third quantile are obtained, respectively, by setting *q* = 0.25, 0.5, and 0.75 in (11). It’s simple to simulate the random variable. The random variable *Y* = *y*_*q*_ at *q* follows (5), if *Q* is a uniform variate (0, 1).

### 3.4 Incomplete moments

The UEL distribution’s s^*th*^ lower IM, is given by:

$$\eta_{s}\left(t\right)=\lambda \delta \vartheta \int_{0}^{t}y^{s-1}{\left(1-\lambda \;\text{ln}\left(y\right)\right)}^{-\delta -1}{\left\{1-{\left(1-\lambda \;\text{ln}\left(y\right)\right)}^{-\delta }\right\}}^{\vartheta -1}dy.$$

Using binomial expansion and let *z* = −λ ln(*y*), then *η*_*s*_(*t*), can be written as:

$$\eta_{s}\left(t\right)=\sum_{k=0}^{\infty }{\left(-1\right)}^{k}\left(\begin{array}{c} \vartheta -1\\ k \end{array}\right)\delta \vartheta \int_{-\lambda \;\text{ln}\;t}^{\infty }e^{\frac{-sz}{\lambda }}{\left(1+z\right)}^{-\delta \left(k+1\right)-1}dz.$$

Also, we use the exponential expansion, then *η*_*s*_(*t*), is as follows:

$$\eta_{s}\left(t\right)=\sum_{k,j=0}^{\infty }\frac{{\left(-1\right)}^{k+j}\delta \vartheta s^{j}}{j!\lambda^{j}}\left(\begin{array}{c} \vartheta -1\\ k \end{array}\right)\int_{-\lambda \;\text{ln}\;t}^{\infty }z^{j}{\left(1+z\right)}^{-\delta \left(k+1\right)-1}dz.$$

Let *x* = (1 + *z*)^−1^, then *η*_*s*_(*t*), is as follows:

$$\begin{array}{c} \eta_{s}\left(t\right)=\sum_{k,j=0}^{\infty }\frac{{\left(-1\right)}^{k+j}\delta \vartheta s^{j}}{j!\lambda^{j}}\left(\begin{array}{c} \vartheta -1\\ k \end{array}\right)\int_{0}^{{\left(1-\lambda \;\text{ln}\;t\right)}^{-1}}{\left(1-x\right)}^{j}{\left(x\right)}^{\delta \left(k+1\right)-j-1}dx\\ \;\;\;\;\;\;\;\;\;\;=\sum_{k,j=0}^{\infty }\frac{{\left(-1\right)}^{j+k}\delta \vartheta s^{j}}{j!\lambda^{j}}\left(\begin{array}{c} \vartheta -1\\ k \end{array}\right)B[1+j,\delta \left(k+1\right)-j,{\left(1-\lambda \;\text{ln}\;t\right)}^{-1}], \end{array}$$

where B(.,.,t) stands for incomplete BF. The Lorenz curve, defined by *L*(*t*)$=\eta_{1}\left(t\right)/\mu_{1}^{\prime }\;$ is notable applications of the first IM. These curves are particularly important in the fields of economics, demography, insurance, etc.

### 3.5 Information measures

Here, certain uncertainty measures are investigated including Rényi (Ré) entropy, Havrda and Charvat (H-C) entropy, and *ω*− entropy.

The Ré entropy of a random variable represents a measure of the variation of the uncertainty. The Ré entropy of a random variable *Y* has the UEL distribution defined by:

$$\begin{array}{c} E_{R}\left(\omega \right)={\left(1-\omega \right)}^{-1}\;\text{ln}\left(\int_{0}^{1}{\left(f\left(y\right)\right)}^{\omega }dy\right).\;\; \end{array}$$ (12)

Substituting (6) in (12), then

$$\;E_{R}\left(\omega \right)={\left(1-\omega \right)}^{-1}\;\text{ln}\left({\left(\lambda \delta \vartheta \right)}^{\omega }\int_{0}^{1}\;\;\;\;y^{-\omega }{\left(1-\lambda \;\text{ln}\left(y\right)\right)}^{-\omega \left(\delta +1\right)}{\left\{1-{\left(1-\lambda \;\text{ln}\left(y\right)\right)}^{-\delta }\right\}}^{\omega \left(\vartheta -1\right)}dy\right).\;\;$$

Using the binomial expansion, then *E*_*R*_(*ω*) is converted to

$$\;E_{R}\left(\omega \right)={\left(1-\omega \right)}^{-1}\;\text{ln}\left(\sum_{m=0}^{\infty }{\left(-1\right)}^{m}\left(\begin{array}{c} \omega \left(\vartheta -1\right)\\ m \end{array}\right){\left(\lambda \delta \vartheta \right)}^{\omega }\int_{0}^{1}\;\;\;\;y^{-\omega }{\left(1-\lambda \;\text{ln}\left(y\right)\right)}^{-\omega \left(\delta +1\right)-\delta m}dy\right).\;\;$$

Let *z* = −λ ln(*y*), and expand exponentially, then the Ré entropy of the UEL distribution

$$\begin{array}{c} \;\begin{array}{c} E_{R}\left(\omega \right)={\left(1-\omega \right)}^{-1}\;\text{ln}\left(\sum_{m,j=0}^{\infty }\frac{{\left(-1\right)}^{m+j}{\left(1-\omega \right)}^{j}}{j!}\left(\begin{array}{c} \omega \left(\vartheta -1\right)\\ m \end{array}\right)\lambda^{\omega -1}{\left(\delta \vartheta \right)}^{\omega }B\left(j+1,\omega \left(\delta +1\right)+\delta m-j\right)\right). \end{array} \end{array}$$

The H-C entropy measure of the UEL distribution is given by

$$\;\begin{array}{c} H_{R}\;\left(\omega \right)=\frac{1}{2^{1-\omega }-1}[{\left(\int_{0}^{1}{\left(f\left(y\right)\right)}^{\omega }dy\right)}^{\frac{1}{\omega }}-1],\;\;\;\;\;\omega \neq 1,\;\;\;\omega >0\\ \;\;\;\;\;\;\;\;\;\;\;\;\;\;=\frac{1}{2^{1-\omega }-1}[{\left(\sum_{m,j=0}^{\infty }\frac{{\left(-1\right)}^{m+j}{\left(1-\omega \right)}^{j}}{j!}\left(\begin{array}{c} \omega \left(\vartheta -1\right)\\ m \end{array}\right)\lambda^{\omega -1}{\left(\delta \vartheta \right)}^{\omega }B\left(j+1,\omega \left(\delta +1\right)+\delta m-j\right)\right)}^{\frac{1}{\omega }}-1]. \end{array}$$

The *ω*− entropy measure of the UEL distribution is obtained as follows:

$$\;\begin{array}{c} \xi_{R}\;\left(\omega \right)=\frac{1}{\omega -1}[1-\int_{0}^{1}{\left(f\left(y\right)\right)}^{\omega }\;dy],\;\;\;\;\;\omega \neq 1,\;\;\;\omega >0\\ \;\;\;\;\;\;\;\;\;\;\;\;=\frac{1}{\omega -1}[1-\left(\sum_{m,j=0}^{\infty }\frac{{\left(-1\right)}^{m+j}{\left(1-\omega \right)}^{j}}{j!}\left(\begin{array}{c} \omega \left(\vartheta -1\right)\\ m \end{array}\right)\lambda^{\omega -1}{\left(\delta \vartheta \right)}^{\omega }B\left(j+1,\omega \left(\delta +1\right)+\delta m-j\right)\right)]. \end{array}$$

Table 2 gives some numerical values of *E*_*R*_(*ω*), H_*R*_(*ω*), and *ξ*_*R*_(*ω*) for the same selected parameter values provided in Table 1.

**Table 2:** Entropy measures of the UEL distribution.

*ω*	Measures	(a)	(b)	(c)	(d)	(e)	(f)
0.5	*E*_*R*_ (*ω*)	-0.708	-0.497	-2.219	-0.459	0.106	-0.875
	*H*_*R*_ (*ω*)	-1.224	-0.946	-2.152	-0.889	0.269	-1.408
	*ξ*_*R*_ (*ω*)	-0.596	-0.44	-1.34	-0.41	0.109	-0.709
0.9	*E*_*R*_ (*ω*)	-4.723	-0.386	-0.386	-3.817	1.404	-3.893
	*H*_*R*_ (*ω*)	-5.688	-0.585	-0.585	-4.816	2.352	-4.892
	*ξ*_*R*_ (*ω*)	-3.764	-0.379	-0.379	-3.173	1.507	-3.225

### 3.6 Stress–strength model

In statistical literature, the term “S-S reliability” is typically represented as A = *P*[*Y*_2_ < *Y*_1_]. The expression comes from a basic scenario in which a system with random strength *Y*_1_ is exposed to random stress *Y*_2_, with the system failing if the stress exceeds the strength. Let *Y*_1_ and *Y*_2_, are independent random variables with UEL (λ, *δ*, *ϑ*_1_), and UEL (λ, *δ*, *ϑ*_2_) distributions. The S-S reliability of the UEL distribution is then determined as follows:

$$\begin{array}{c} A=1-\int_{0}^{1}\frac{\lambda \delta \vartheta_{1}}{y}{\left(1-\lambda \;\text{ln}\left(y\right)\right)}^{-\delta -1}{\left\{1-{\left(1-\lambda \;\text{ln}\left(y\right)\right)}^{-\delta }\right\}}^{\vartheta_{1}-1}{\left\{1-{\left(1-\lambda \;\text{ln}\left(y\right)\right)}^{-\delta }\right\}}^{\vartheta_{2}}dy. \end{array}$$ (13)

Then S-S reliability is obtained as follows

$$A=1-\frac{\vartheta_{1}}{\vartheta_{2}+\vartheta_{1}}=\frac{\vartheta_{2}}{\vartheta_{2}+\vartheta_{1}}.$$

## 4 Parameter estimation of the UEL model

The parameter estimators of the UEL distribution based on ML, MPS, and Bayesian estimation methods are discussed in this section.

### 4.1 ML method

Let *y*_1_,…,*y*_*n*_ be the observed samples from the UEL distribution with parameters *δ*, λ, and *ϑ*. The likelihood function (LF), say *L* (*y*|Θ) of the UEL distribution is expressed as:

$$\begin{array}{c} L\left(\underline{y}|\Theta \right)=\lambda^{n}\delta^{n}\vartheta^{n}\prod_{i=1}^{n}\frac{{\left(1-\lambda \;\text{ln}\left(y_{i}\right)\right)}^{-\delta -1}}{y_{i}}{\left\{1-{\left(1-\lambda \;\text{ln}\left(y_{i}\right)\right)}^{-\delta }\right\}}^{\vartheta -1}. \end{array}$$ (14)

Then the ln of LF, say *ℓ*, of the UEL distribution is:

$$\begin{array}{c} \;\ell =n\;\text{ln}\;[\lambda \delta \vartheta ]-\sum_{i=1}^{n}\;\text{ln}\;\left(y_{i}\right)-\left(\delta +1\right)\sum_{i=1}^{n}\;\text{ln}\;[1-\lambda \;\text{ln}\;\left(y_{i}\right)]+\left(\vartheta -1\right)\sum_{i=1}^{n}\;\text{ln}\;\left\{1-{\left[1-\lambda \;\text{ln}\left(y_{i}\right)\right]}^{-\delta }\right\}. \end{array}$$ (15)

The ML equations, which are based on (15), are therefore as follows:

$$\;\frac{\partial \ell }{\partial \delta }=\;\frac{n}{\delta }-\sum_{i=1}^{n}\text{ln}\;[1-\lambda \;\text{ln}\left(y_{i}\right)]+\left(\vartheta -1\right)\sum_{i=1}^{n}\frac{\text{ln}\;[1-\lambda \;\text{ln}\left(y_{i}\right)]}{{\left[1-\lambda \;\text{ln}\left(y_{i}\right)\right]}^{\delta }-1},$$

$$\frac{\partial \ell }{\partial \vartheta }=\;\frac{n}{\vartheta }+\sum_{i=1}^{n}\text{ln}\;\left\{1-{\left[1-\lambda \;\text{ln}\left(y_{i}\right)\right]}^{-\delta }\right\},$$

$$\frac{\partial \ell }{\partial \lambda }=\;\frac{n}{\lambda }+\left(\delta +1\right)\sum_{i=1}^{n}\frac{\text{ln}\left(y_{i}\right)}{1-\lambda \;\text{ln}\left(y_{i}\right)}-\sum_{i=1}^{n}\frac{\left(\vartheta -1\right)\delta \;{\left[1-\lambda \;\text{ln}\left(y_{i}\right)\right]}^{-\delta -1}\text{ln}\left(y_{i}\right)}{1-{\left[1-\lambda \;\text{ln}\left(y_{i}\right)\right]}^{-\delta }}.$$

Solving the non-linear equations *∂ℓ*/*∂δ* = 0, *∂ℓ*/*∂*λ = 0, and *∂ℓ*/*∂ϑ* = 0, numerically using optimization algorithm as conjugate-gradient optimization, the ML estimators of *δ*, λ, and *ϑ* are obtained.

### 4.2 MPS method

The MPS technique, which is an alternative to the ML methodology, offers a parameter estimate of a continuous distribution. Suppose that *y*_(1)_,…,*y*_(*n*)_ be the observed ordered samples from the UEL distribution with parameters *δ*, λ, and *ϑ*. The MPS estimators of *δ*, λ, and *ϑ* are generated by maximizing the following:

$$\ell \left(g\right)=\frac{1}{n+1}\left\{\begin{array}{c} \sum_{i=2}^{n}\text{ln}\left({\left\{1-{\left[1-\lambda \;\text{ln}\left(y_{\left(i\right)-1}\right)\right]}^{-\delta }\right\}}^{\vartheta }-{\left\{1-{\left[1-\lambda \;\text{ln}\left(y_{\left(i\right)}\right)\right]}^{-\delta }\right\}}^{\vartheta }\right)+\\ \text{ln}\left(1-{\left\{1-{\left[1-\lambda \;\text{ln}\left(y_{\left(1\right)}\right)\right]}^{-\delta }\right\}}^{\vartheta }\right)+\vartheta \;\text{ln}\left\{1-{\left[1-\lambda \;\text{ln}\left(y_{\left(n\right)}\right)\right]}^{-\delta }\right\} \end{array}\right\}.$$

Solving the non-linear equations *∂ℓ*(*g*)/*∂δ* = 0, *∂ℓ*(*g*)/*∂*λ = 0, and *∂ℓ*(*g*)/*∂ϑ* = 0 via numerical technique, the MPS estimators of *δ*, λ, and *ϑ* are provided. The MPS estimators are derived by partly differentiating the natural logarithm of the UEL distribution’s product spacing function with respect to population parameters and using an optimization algorithm (conjugate-gradient or Newton-Raphson optimization).

### 4.3 Bayesian method

Here, the Bayesian estimator of the UEL parameters is produced. The Bayesian estimator is regarded under symmetric (squared error loss function (SELF)) which is defined as

$$L\left(\tilde{\delta },\delta \right)=E{\left(\tilde{\delta }-\delta \right)}^{2},\;\;L\left(\tilde{\vartheta },\vartheta \right)=E{\left(\tilde{\vartheta }-\vartheta \right)}^{2},L\left(\tilde{\lambda },\lambda \right)=E{\left(\tilde{\lambda }-\lambda \right)}^{2}.$$

Assuming that the prior distribution of *δ*, λ, and *ϑ*, denoted by *π*(*δ*), *π*(*ϑ*), *π*(λ), have an independent gamma distribution. The joint gamma prior density of *δ*, λ and *ϑ* can be written as:

$$\begin{array}{c} \pi \left(\delta ,\lambda ,\vartheta \right)\propto \delta^{a_{1}-1}e^{-s_{1}\delta }\lambda^{a_{2}-1}e^{-s_{2}\lambda }\vartheta^{a_{3}-1}e^{-s_{3}\vartheta };a_{1},s_{1},a_{2},s_{2},a_{3},s_{3}>0. \end{array}$$ (16)

From (14) and (16), the joint posterior of the UEL distribution with parameters *δ*, λ, and *ϑ* is

$$\begin{array}{c} \pi \left(\delta ,\lambda ,\vartheta |\underline{y}\right)\propto \lambda^{n+a_{2}-1}\delta^{n+a_{1}-1}\vartheta^{n+a_{3}-1}e^{-\delta \left\{s_{1}+\text{ln}\left[1-\lambda \;\text{ln}\left(y_{i}\right)\right]\right\}}e^{-\vartheta \left(s_{3}-\text{ln}\left\{1-{\left(1-\lambda \;\text{ln}\left(y_{i}\right)\right)}^{-\delta }\right\}\right)}e^{-s_{2}\lambda }\\ \;\;\;\;\;\;\;\;\;\;\;\;\;\;\;\;\;\;\;\;\;\;\;\;\;\;\;\prod_{i=1}^{n}{\left(1-\lambda \;\text{ln}\left(y_{i}\right)\right)}^{-1}{\left\{1-{\left(1-\lambda \;\text{ln}\left(y_{i}\right)\right)}^{-\delta }\right\}}^{-1}. \end{array}$$

Bayesian estimators may be produced using the Markov Chain Monte Carlo (MCMC) technique. Gibbs sampling, as well as the more generic Metropolis within Gibbs samplers, are important MCMC techniques. The Metropolis-Hastings (MH) algorithm and Gibbs sampling are two well-known applications of the MCMC approach. To produce random samples from conditional posterior densities of *δ*, λ and *ϑ* as follows:

$$\pi \left(\delta |\lambda ,\vartheta ,\underline{y}\right)\propto \delta^{n+a_{1}-1}e^{-\delta \left\{s_{1}+\sum_{i-1}^{n}\text{ln}\left[1-\lambda \;\text{ln}\left(y_{i}\right)\right]\right\}}\prod_{i=1}^{n}{\left\{1-{\left(1-\lambda \;\text{ln}\left(y_{i}\right)\right)}^{-\delta }\right\}}^{\vartheta -1},$$

$$\begin{array}{c} \begin{array}{cc} \pi \left(\vartheta |\delta ,\lambda ,\underline{y}\right) & \propto \vartheta^{n+a_{3}-1}e^{-\vartheta \left(s_{3}-\sum_{i=1}^{n}\text{ln}\left\{1-{\left(1-\lambda \;\text{ln}\left(y_{i}\right)\right)}^{-\delta }\right\}\right)}\;\\ & ∼Gamma\left(n+a_{3},1/1\left(s_{3}-\sum_{i=1}^{n}\;\text{ln}\;\left\{1-{\left(1-\lambda \;\text{ln}\left(y_{i}\right)\right)}^{-\delta }\right\}\right)\;\left(s_{3}-\sum_{i=1}^{n}\text{ln}\left\{1-{\left(1-\lambda \;\text{ln}\left(y_{i}\right)\right)}^{-\delta }\right\}\right)\right), \end{array} \end{array}$$

$$\pi \left(\lambda |\delta ,\vartheta ,\underline{y}\right)\propto \lambda^{n+a_{2}-1}e^{-s_{2}\lambda }\prod_{i=1}^{n}{\left(1-\lambda \;\text{ln}\left(y_{i}\right)\right)}^{-\delta -1}{\left\{1-{\left(1-\lambda \;\text{ln}\left(y_{i}\right)\right)}^{-\delta }\right\}}^{\vartheta -1}.$$

The Bayesian estimators are obtained via SELF.

## 5 Simulation study

A Monte-Carlo simulation study was conducted to analyze and compare the behaviour of different estimates methods based on mean squared errors (MSEs) and biases.

From the UEL distribution, generate 5000 random samples of sizes *n* = 30, 70, and 150. Various actual parameter values were taken into account as follows: In Table 3: (*δ*, λ, *ϑ*) = (0.5, 0.5, 0.5), (0.5, 0.5, 2.5), (0.5, 2.5, 0.5), and (0.5, 2.5, 2.5). In Table 4: (*δ*, λ, *ϑ*) = (2.5, 0.5, 0.5), (2.5, 0.5, 2.5), (2.5, 2.5, 0.5), and (2.5, 2.5, 2.5). Since none of the proposed estimators for any unknown parameter can be solved analytically, we can only numerically evaluate them using computing resources and numerical techniques. Because of this, the ML and MPS are calculated using the ‘maxLik’ package, which employs the Newton-Raphson method of maximization in the computations. Additionally, the ‘CODA’ package, which analyses MCMC outputs and diagnoses lack of convergence, is used to compute Bayesian estimation.

**Table 3:** The UEL distribution biases and MSEs under various estimation methods at *δ* = 0.5.

*δ* = 0.5				ML					MPS			Bayesian		
λ	*ϑ*	n		Bias	MSE	L.CI	L.BP	L.BT	Bias	MSE	L.CI	Bias	MSE	L.CI
0.5	0.5	30	*δ*	0.4152	0.3473	1.6403	0.0491	0.0480	0.2261	0.1456	1.3946	0.1797	0.0698	0.6726
			λ	0.0922	0.6355	3.1055	0.0975	0.0962	0.0107	0.2771	2.1456	0.1348	0.1092	1.0709
			*ϑ*	0.0705	0.0617	0.9344	0.0297	0.0296	-0.0129	0.0287	0.7462	0.0741	0.0219	0.4026
		70	*δ*	0.3224	0.1546	0.8821	0.0270	0.0270	0.2325	0.0889	0.8220	0.1431	0.0335	0.4141
			λ	-0.1056	0.1631	1.5289	0.0464	0.0469	-0.1249	0.1114	1.2331	0.0828	0.0671	0.7367
			*ϑ*	0.0072	0.0161	0.4971	0.0167	0.0163	-0.0280	0.0121	0.4530	0.0433	0.0069	0.2531
		150	*δ*	0.2814	0.1028	0.6034	0.0188	0.0188	0.2372	0.0743	0.5708	0.1257	0.0205	0.2636
			λ	-0.1678	0.0733	0.8338	0.0267	0.0268	-0.1731	0.0651	0.7409	0.0244	0.0165	0.3862
			*ϑ*	-0.0120	0.0060	0.2998	0.0101	0.0100	-0.0297	0.0058	0.2924	0.0239	0.0022	0.1478
	2.5	30	*δ*	0.4687	0.2584	0.7725	0.0257	0.0252	0.3669	0.1634	0.7671	0.1404	0.1747	0.6166
			λ	-0.3034	0.1198	0.6529	0.0222	0.0218	-0.3010	0.1179	0.6460	0.1206	0.0913	1.0269
			*ϑ*	-0.2102	0.5030	2.6565	0.0891	0.0882	-0.5149	0.5660	2.6832	0.0901	0.4216	1.0629
		70	*δ*	0.4341	0.2095	0.5685	0.0197	0.0190	0.3859	0.1623	0.5215	0.1308	0.1517	0.3235
			λ	-0.3258	0.1173	0.4141	0.0124	0.0125	-0.3321	0.1019	0.3808	0.1022	0.0841	0.2988
			*ϑ*	-0.3449	0.3839	2.0189	0.0673	0.0653	-0.5134	0.4811	1.9977	0.0821	0.2578	0.9699
		150	*δ*	0.4174	0.1852	0.4111	0.0128	0.0126	0.3926	0.1615	0.3607	0.0825	0.0940	0.3014
			λ	-0.3384	0.1018	0.2363	0.0074	0.0072	-0.3424	0.0912	0.2161	0.0783	0.0528	0.1470
			*ϑ*	-0.4016	0.3016	1.4688	0.0448	0.0456	-0.4875	0.3551	1.4298	0.0737	0.1973	0.9304
2.5	0.5	30	*δ*	0.3219	0.2720	1.6091	0.0490	0.0482	0.1212	0.0880	1.2625	0.1538	0.0812	0.7496
			λ	-0.3868	2.1097	5.4909	0.1756	0.1743	-0.4145	1.1050	3.8164	0.3763	1.1706	3.6830
			*ϑ*	0.0317	0.0292	0.6591	0.0195	0.0203	-0.0332	0.0169	0.5576	0.0921	0.0288	0.5577
		70	*δ*	0.1899	0.0743	0.7675	0.0235	0.0236	0.1170	0.0334	0.6226	0.1493	0.0681	0.5865
			λ	-0.3971	0.8703	3.3109	0.0983	0.0997	-0.4435	0.7266	2.9012	0.3708	0.7238	2.2403
			*ϑ*	0.0196	0.0083	0.3490	0.0115	0.0115	-0.0149	0.0073	0.3653	0.0968	0.0065	0.4692
		150	*δ*	0.1741	0.0442	0.4623	0.0149	0.0146	0.1176	0.0208	0.3853	0.0988	0.0164	0.2667
			λ	-0.5348	0.8677	2.9911	0.0962	0.0958	-0.4328	0.5013	2.0957	0.1593	0.5347	2.8764
			*ϑ*	0.0022	0.0045	0.2641	0.0081	0.0082	-0.0083	0.0038	0.2509	0.0198	0.0026	0.1671
	2.5	30	*δ*	0.2951	0.1311	0.8228	0.0252	0.0254	0.1736	0.0512	0.6902	0.1414	0.0249	0.2669
			λ	-0.7684	1.7003	4.1318	0.1256	0.1262	-0.7139	0.9590	2.5745	-0.0118	0.1278	1.4368
			*ϑ*	0.3530	1.3779	4.3907	0.1352	0.1352	-0.0597	0.5152	3.2180	0.2867	0.1785	1.2076
		70	*δ*	0.2264	0.0679	0.5066	0.0162	0.0162	0.1961	0.0474	0.4024	0.1605	0.0239	0.2132
			λ	-0.6426	0.6977	2.0930	0.0664	0.0674	-0.6674	0.6538	2.0422	-0.1455	0.1156	1.4005
			*ϑ*	0.3519	0.4282	2.1638	0.0730	0.0723	-0.0103	0.3210	2.5837	0.3660	0.1623	1.2026
		150	*δ*	0.2399	0.0669	0.4141	0.0126	0.0125	0.1925	0.0403	0.2708	0.1710	0.0231	0.1685
			λ	-0.9900	0.4474	2.6811	0.0839	0.0830	-0.5890	0.3962	1.5447	-0.2859	0.1021	1.3820
			*ϑ*	-0.0151	0.3479	2.3124	0.0705	0.0702	0.0181	0.1699	1.5818	0.3642	0.1522	1.1361

**Table 4:** The UEL distribution biases and MSEs under various estimation methods at *δ* = 2.5.

*δ* = 2.5,				ML					MPS			Bayesian		
λ	*ϑ*	n		Bias	MSE	L.CI	L.BP	L.BT	Bias	MSE	L.CI	Bias	MSE	L.CI
0.5	0.5	30	*δ*	0.2534	0.8939	3.5725	0.1149	0.1147	-0.2271	0.5842	3.3428	0.0097	0.0048	0.2552
			λ	0.2051	0.4972	2.6458	0.0868	0.0852	0.1725	0.2931	2.0453	0.1540	0.1004	0.9263
			*ϑ*	0.0511	0.0269	0.6118	0.0194	0.0197	-0.0204	0.0154	0.5513	0.0610	0.0231	0.5159
		70	*δ*	0.0607	0.4069	2.4905	0.0790	0.0791	-0.2022	0.3633	2.4898	-0.0021	0.0047	0.2318
			λ	0.1265	0.1981	1.6737	0.0511	0.0514	0.1211	0.1273	1.3216	0.1063	0.0479	0.6829
			*ϑ*	0.0273	0.0093	0.3629	0.0114	0.0114	-0.0075	0.0066	0.3513	0.0368	0.0088	0.3270
		150	*δ*	0.0994	0.3791	2.3831	0.0765	0.0757	-0.1373	0.2480	2.1140	-0.0073	0.0043	0.2234
			λ	0.0507	0.0592	0.9331	0.0313	0.0310	0.0702	0.0493	0.8069	0.0650	0.0203	0.4716
			*ϑ*	0.0074	0.0032	0.2182	0.0072	0.0070	-0.0088	0.0029	0.2233	0.0159	0.0031	0.2162
	2.5	30	*δ*	0.2073	0.7242	3.2370	0.1077	0.1051	-0.1477	0.5495	3.2041	-0.0021	0.0076	0.3390
			λ	0.2210	0.4715	2.5497	0.0851	0.0843	0.1785	0.2425	1.8426	0.0806	0.0253	0.4886
			*ϑ*	0.6106	2.0581	5.0914	0.1629	0.1614	-0.0375	0.6589	3.8282	0.1827	0.1629	1.3451
		70	*δ*	0.0577	0.3239	2.2206	0.0673	0.0671	-0.0679	0.2915	2.2264	-0.0101	0.0072	0.3260
			λ	0.1221	0.1792	1.5896	0.0535	0.0523	0.0686	0.0814	1.1399	0.0618	0.0150	0.3877
			*ϑ*	0.3319	1.0365	3.7747	0.1229	0.1200	-0.0573	0.3420	2.6717	0.1795	0.1572	1.2441
		150	*δ*	0.0728	0.2567	1.9663	0.0621	0.0619	-0.0517	0.1872	1.8093	-0.0140	0.0061	0.4476
			λ	0.0572	0.0747	1.0483	0.0331	0.0334	0.0444	0.0399	0.7762	0.0543	0.0106	0.3254
			*ϑ*	0.1506	0.3518	2.2499	0.0719	0.0702	-0.0351	0.1773	1.8312	0.1633	0.1400	1.1329
2.5	0.5	30	*δ*	0.3782	1.1126	3.8617	0.1172	0.1171	-0.2332	0.4344	3.0291	0.0272	0.0053	0.2658
			λ	0.1428	1.5117	4.7895	0.1578	0.1562	0.0452	0.4970	2.8567	0.2498	0.2786	1.7161
			*ϑ*	0.0372	0.0205	0.5423	0.0167	0.0161	-0.0341	0.0134	0.5056	0.0428	0.0146	0.4139
		70	*δ*	0.1876	0.5000	2.6740	0.0821	0.0834	-0.1496	0.2065	2.0201	0.0160	0.0063	0.2993
			λ	0.0628	0.7602	3.4108	0.0985	0.1011	0.0249	0.2329	1.9280	0.2064	0.2057	1.5434
			*ϑ*	0.0153	0.0064	0.3067	0.0092	0.0091	-0.0212	0.0051	0.3041	0.0245	0.0056	0.2631
		150	*δ*	0.1727	0.3771	2.3112	0.0742	0.0756	-0.0861	0.0866	1.3626	0.0154	0.0065	0.3105
			λ	0.0145	0.4360	2.5890	0.0810	0.0808	0.0348	0.0996	1.2103	0.1742	0.1554	1.3675
			*ϑ*	0.0094	0.0029	0.2064	0.0063	0.0063	-0.0095	0.0024	0.2091	0.0160	0.0028	0.1876
	2.5	30	*δ*	0.7699	2.7260	5.7282	0.1766	0.1754	-0.0272	0.5209	3.6256	0.0167	0.0068	0.3157
			λ	0.3081	4.2560	8.0003	0.2467	0.2461	0.0805	0.9764	4.0902	0.1762	0.1374	1.2469
			*ϑ*	0.4486	1.8452	5.0285	0.1581	0.1581	-0.2101	0.6130	3.6167	0.1311	0.1111	1.1672
		70	*δ*	0.3692	1.0601	3.7696	0.1186	0.1189	-0.0299	0.2531	2.3688	0.0137	0.0066	0.3127
			λ	0.3152	3.0336	6.7182	0.2173	0.2169	0.0352	0.5299	3.1316	0.1517	0.1139	1.1526
			*ϑ*	0.2459	0.9169	3.6296	0.1141	0.1138	-0.1560	0.2706	2.3480	0.1175	0.1038	1.1546
		150	*δ*	0.2170	0.4631	2.5295	0.0798	0.0800	-0.0231	0.1121	1.5503	0.0064	0.0061	0.3062
			λ	0.0239	0.8757	3.6689	0.1176	0.1175	0.0260	0.2576	1.9861	0.1555	0.0966	1.0547
			*ϑ*	0.0765	0.2538	1.9530	0.0628	0.0618	-0.0773	0.1342	1.5584	0.1190	0.0939	1.0723

**Fig 2:**
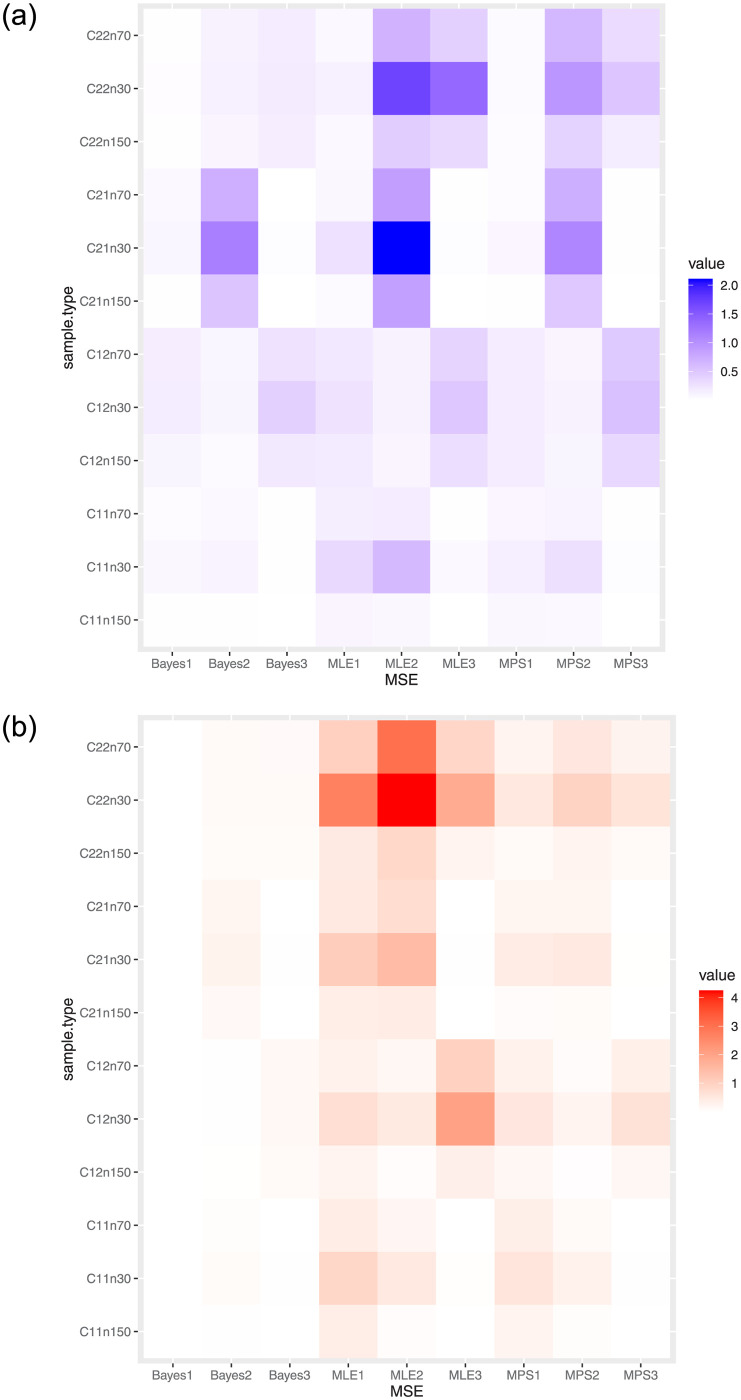
Heatmaps of MSE values of different cases and different estimates: Left at *δ* = 0.5 & right at *δ* = 2.5.

The ML estimate (MLE), MPS estimate (MPSE), and Bayesian estimate of *δ*, λ and *ϑ* are calculated. Then, the biases and MSEs of the different estimates were determined as well as confidence intervals (CIs) were obtained. In MLE, the CIs by asymptotic CIs and bootstrap CIs with different algorithms as bootstrap-P (BP) and bootstrap-T (BT) were calculated. In MPS the confidence lengths (CLs) by asymptotic CIs are calculated. While in the Bayesian estimation method, the credible CIs (CCIs) by the highest posterior density CLs are obtained (see [32, 33]). Simulated results of bias, MSE, and length of CI (L.CI) were scheduled in Tables 3 and 4, and Fig 2.

Some concluding remarks are noticed as the following:

1. As *n* increases, the bias and MSE across all estimates decrease.
2. The measures of MPSE are better than MLE.
3. MLEs and MPSEs are not as good as the metrics of Bayesian estimates.
4. The length of asymptotic CLs for MPSE has smaller values than the length of asymptotic CLs for MLEs.
5. The length of credible CLs for Bayesian has smaller values than the length of asymptotic CLs for MLE and MPSE.
6. The length of bootstrap CLs gets the smallest value of the length of CIs.
7. The length of bootstrap-T CLs gets the smallest value of the length of bootstrap-P CIs.

## 6 The UEL quantile regression model

According to the QF of the UEL distribution in Eq (11), we shall re-parametrize (5) in terms of the *μ*^*th*^ quantile *μ* = *y*(*q*; *δ*, λ, *ϑ*) such that λ can be written as follows:

$$\lambda =\frac{1-{\left(1-{\left(1-q\right)}^{\frac{1}{\vartheta }}\right)}^{-1/\delta }}{\text{ln}\left(\mu \right)},\;\;\;\;0<q<1.$$

The PDF and CDF of the UEL distribution can be stated as follows with this parametrzation

$$\begin{array}{c} \;f\left(y;\Theta \right)=\frac{1-{\left(A\left(q,\vartheta \right)\right)}^{-1/\delta }}{\text{ln}\left(\mu \right)}\frac{\delta \vartheta }{y}{\left(1-\frac{1-{\left(A\left(q,\vartheta \right)\right)}^{-1/\delta }}{\text{ln}\left(\mu \right)}\text{ln}\left(y\right)\right)}^{-\delta -1}{\left\{1-{\left(1-\frac{1-{\left(A\left(q,\vartheta \right)\right)}^{-1/\delta }}{\text{ln}\left(\mu \right)}\text{ln}\left(y\right)\right)}^{-\delta }\right\}}^{\vartheta -1},\; \end{array}$$ (17)

where $A\left(q,\vartheta \right)=1-{\left(1-q\right)}^{\frac{1}{\vartheta }}$ *δ*, *ϑ* > 0, 0 < *μ* < 1, 0 < *y* < 1 and 0 < *q* < 1, and the CDF is

$$F\left(y;\Theta \right)\;\;\;\;=1-{\left\{1-{\left(1-\frac{1-{\left(A\left(q,\vartheta \right)\right)}^{-1/\delta }}{\text{ln}\left(\mu \right)}\text{ln}\left(y\right)\right)}^{-\delta }\right\}}^{\vartheta },\;\;\;\;\;\;\;\;\;\;\;\;\;\;\;\;\delta ,\vartheta >0,\;\;0<\mu <1,\;0\;<y<1.$$

The notation *Y* ∼ UEL(*δ*, *ϑ*, *μ*) will be used from now on, where *μ* ∈ (0, 1) is the quantile parameter, *δ*, and *ϑ* are the shape parameters, and *q* ∈ (0, 1) is presumed to be known. The behavior of (17) can be investigated using the logarithmic scale for various values of *δ*, *ϑ*, *μ* and *q*.

$$\;\frac{\partial \;\text{ln}\;\left[f\left(y;\Theta \right)\right]}{\partial y}=-\frac{1}{y}+\frac{\left(\delta +1\right)}{\text{ln}\left(\mu \right)}\frac{1-{\left(A\left(q,\vartheta \right)\right)}^{-1/\delta }}{\left[1-\hbar_{y}\left(\delta ,\mu ,q,\vartheta \right)\right]y}-\frac{\delta \left(\vartheta -1\right)}{\text{ln}\left(\mu \right)}\frac{{\left(1-\hbar_{y}\left(\delta ,\mu ,q,\vartheta \right)\right)}^{-\delta -1}\left(1-{\left(A\left(q,\vartheta \right)\right)}^{-1/\delta }\right)}{\left[1-{\left(1-\hbar_{y}\left(\delta ,\mu ,q,\vartheta \right)\right)}^{-\delta }\right]y},$$

where $\hbar_{y}\left(\delta ,\mu ,q,\vartheta \right)=\frac{1-{\left(1-{\left(1-q\right)}^{\frac{1}{\vartheta }}\right)}^{-1/\delta }}{\text{ln}\left(\mu \right)}\text{ln}\left(y\right)$, cannot be solved analytically in *y*. We can create a quantile regression model using the re-parametrized PDF (17), as in Ref. [34], where they used the Kumaraswamy distribution and unit Weibull regression model.

Let *Y*_1_,…,*Y*_*n*_ be *n* independent random variables, where each *Y*_*i*_, *i*=1,… *n* follows the PDF (17) with *μ*_*i*_ as unknown quantile parameter, and *δ*, *ϑ* (unknown shape parameter), where *q* ∈ (0, 1) is assumed to be known, i.e, *Y*_*i*_ ∼ *UEL*(*δ*, *ϑ*, *μ*_*i*_). The quantile *μ*_*i*_ of *Y*_*i*_ must satisfy the following functional relation in order for the UEL quantile regression model to be defined here:

$$g\left(\mu_{i}\right)=B^{T}X_{i},\;\;i=1,2,,...,n,$$

where *X*_*i*_ = (1, *X*_1*i*_, …, *X*_(*p*−1)*i*_) represents the observations on *p* known covariates and *B* = (*B*_0_, *B*_1_, …, *B*_*p*−1_)^*T*^ is a p-dimensional vector of unknown regression coefficients, *p* < *n*. We will suppose that the quantile link function g(.) maps (0, 1) into R and is strictly growing and twice differentiable. There are several choices for the link function g(.). For instance, the most popular link functions are:

1. logit: gμi=ln(μi1−μi);
2. probit: *g* (*μ*_*i*_) = Φ^−1^ (*μ*_*i*_); where Φ^−1^ (.) is the standard normal QF;
3. complementary log-log: *g* (*μ*_*i*_) = ln [−ln (1 − *μ*_*i*_)].

We only consider the logit as a link function in this paper because the parameters are directly translated into odds. Reference [35] gave their interpretation for logit when is the beta distribution’s mean. Then, we can write *μ*_*i*_ under the logit link function as follows:

$$\mu_{i}=\frac{e^{B^{T}X_{i}}}{1+e^{B^{T}X_{i}}},\;\;i=1,2,...,n.$$

For assigned *q* ∈ (0, 1), let Θ = (*B*^*T*^, *δ*, *ϑ*) be the vector of *p* unknown parameters to be estimated using the approach of ML. Using the structure of the PDF (17), the log- LF is given by:

$$\;\begin{array}{c} l\left(\Theta \right)=\;n\left\{\text{ln}\left[1-{(A\left(q,\vartheta \right)}^{-1/\delta }\right]\;+\text{ln}\left(\delta \right)+\text{ln}\left(\vartheta \right)\right\}\;\;\;-\sum_{i=1}^{n}\;\text{ln}\;\left(y_{i}\right)\;-\left(\delta +1\right)\sum_{i=1}^{n}\;\text{ln}\;\left(1-\frac{1-{(\left(A\left(q,\vartheta \right)\right)}^{-1/\delta }}{\text{ln}\left(\mu_{i}\right)}\text{ln}\left(y_{i}\right)\right)\;\\ \;\;\;\;\;\;\;\;\;\;\;\;\;\;\;\;\;\;\;\;+\left(\vartheta -1\right)\sum_{i=1}^{n}\;\text{ln}\;\left\{1-{\left(1-\frac{1-{(\left(A\left(q,\vartheta \right)\right)}^{-1/\delta }}{\text{ln}\left(\mu_{i}\right)}\text{ln}\left(y_{i}\right)\right)}^{-\delta }\right\}-\sum_{i=1}^{n}\;\text{ln}\;\left[\text{ln}\left(\mu_{i}\right)\right],\; \end{array}$$

The MLE of parameters cannot be computed analytically and must be calculated numerically using an optimization algorithm such as Newton–Raphson or quasi-Newton. Under regularity conditions and when *n* is large, the asymptotic distribution of the MLE is approximately multivariate normal with mean vector (*B*^*T*^, *δ*, *ϑ*) and *V*^−1^ (Θ) is the variance-covariance matrix, where

$$V^{-1}\left(\Theta \right)=E[-\partial^{2}l\left(\Theta \right)/\partial \Theta \partial \Theta ],$$

is the expected Fisher information matrix.

## 7 Data applications

This section contains three real-world data sets that demonstrate the UEL distribution’s modelling capabilities. Quantile regression modelling is addressed in the first and second data sets, while data modelling is addressed in the third data. Unit-Weibull regression (UWR), beta regression (BetaR) model, original linear regression (OLR) and quantile regression (QR) are three competitor regression models that are compared to the UEL quantile regression model for the first and second data. The QR has been obtained by “rq” function in “quantreg” package in the R program (see [36])). To compare between the UEL and the considered regression models, we compute Akaike’s information criterion (AIC), and Bayesian information criterion (BIC). For the third data set, the fits of the UEL distribution are compared with some other competitive models to illustrate the potentiality of the UEL model. The MLEs and their standard errors (SEs) are computed. The AIC, BIC, corrected AIC (CAIC), Hannan and Quinn information criteria (HQIC), Cramér–von Mises criterion (CVMC), Anderson-Darling criterion (ADC), and Kolmogorov-Smirnov distance (KSD) statistics, as well as their related P-values, were chosen as criteria measures for the third data set.

### 7.1 Quantile regression modeling for confidence of mock jurors in their verdicts

Data with responses of naive mock jurors to the conventional two-option verdict (guilt vs. acquittal) versus a three-option verdict setup (the third option was the Scottish ‘not proven’ alternative), in the presence/absence of conflicting testimonial evidence. A data frame containing 104 observations on three variables. The source of this data is Ref. [37] The following covariates are connected to this response variable Verdict: *x*_1_ is a factor indicating whether a two-option or three-option verdict is requested, where two-option is -1, and three-option is 1. Conflict: *x*_2_ is a factor. Is there conflicting testimonial evidence? If no is 1, yes is -1. The regression framework assumed for *μ*_*i*_ is provided by Logit *μ*_*i*_ = *B*_0_ + *B*_1_*x*_1*i*_ + *B*_2_*x*_2*i*_, *i* = 1, …, 104, where *μ*_*i*_ denotes the median *q* = 0.5 in the UEL regression (UELR) model. Table 5 gives the MLEs and SEs along with the AIC and BIC measures for the UELR, UWR, BetaR, OLR, and QR models.

**Table 5:** The MLE and SE with AIC and BIC for different models for mock jurors data.

	UELR		UWR		BetaR		OLR		QR	
	Estimates	SE	Estimates	SE	Estimates	SE	Estimates	SE	Estimates	SE
*δ*	4.7238	2.4264								
*ϑ*	0.9504	0.1411	0.8514	0.0630	0.8507	0.1092				
*B*_0_	1.2558	0.1445	1.2043	0.1537	0.0771	0.1016	0.7174	0.0209	0.74800	0.0851
*B*_1_	0.1621	0.1371	0.2328	0.1369	0.0995	0.1017	0.0117	0.0209	0.02450	0.0058
*B*_2_	0.0480	0.1411	-0.0368	0.1370	2.6174	0.3307	0.0230	0.0209	0.02450	0.0471
AIC	-56.5601		-54.9753		-50.1073		-21.4312		-23.8917	
BIC	-43.3382		-43.3178		-39.5297		-10.8537		-15.9586	

### 7.2 Quantile regression modeling for proportion of household income spent on food

Here, the proposed data on the proportion of income spent on food for a random sample of 38 households in a large US city. The source of this data is Ref. [38]. The covariates connected to this response variable are Income: *x*_1_ is household income Persons: *x*_2_ is the number of persons living in the household. The dependent variable is I(food/income). The regression pattern assumed for *μ*_*i*_ is as below Logit *μ*_*i*_ = *B*_0_ + *B*_1_*x*_1*i*_ + *B*_2_*x*_2*i*_, *i* = 1, …, 38 where *μ*_*i*_ denotes the median *q* = 0.5 in the UELR model. Table 6 gives the MLEs and SEs along with the AIC and BIC measures for the UELR, UWR, BetaR, OLR, and QR models. We note that UELR has a smaller value of AIC, and BIC than UWR, BetaR, OLR, and QR models, then the UELR model is better than UWR, BetaR, OLR, and QR models.

**Table 6:** The MLE and SE with AIC and BIC for different models for food expenditure data.

Food Expenditure	UELR		UWR		BetaR		OLR		QR	
	Estimates	SE	Estimates	SE	Estimates	SE	Estimates	SE	Estimates	SE
*δ*	39.6223	11.1734	4.5610	0.5416	-0.6225	0.2239				
*ϑ*	350.0053	28.0688								
*B*_0_	-0.6986	0.2199	-0.4948	0.2308	-0.0123	0.0030	0.3417	0.0488	0.34733	0.05065
*B*_1_	-0.0129	0.0027	-0.0131	0.0041	0.1185	0.0353	-0.0025	0.0006	-0.00193	0.00056
*B*_2_	0.1544	0.0330	0.0796	0.0473	35.6098	8.0796	0.0258	0.0076	0.01862	0.00396
AIC	-84.3178		-74.3418		-82.6670		-80.7407		-82.11628	
BIC	-76.1298		-67.7915		-76.1167		-74.1903		-76.1202	

**Fig 3:**
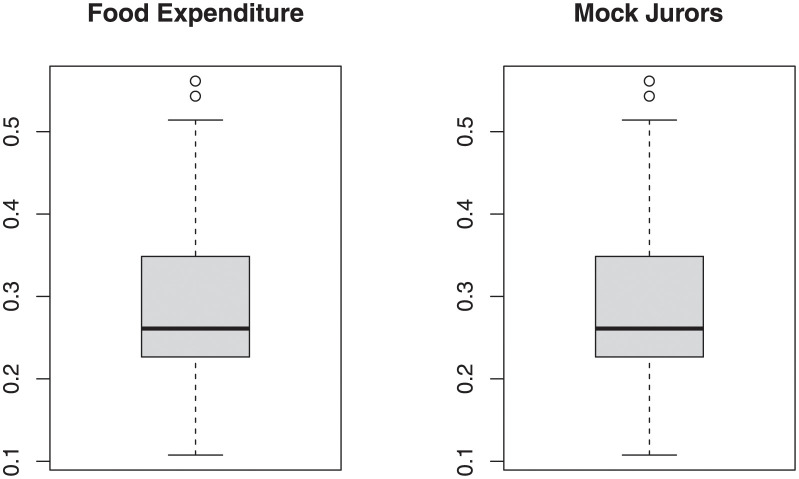
Boxplot for dependent variables.

**Fig 4:**
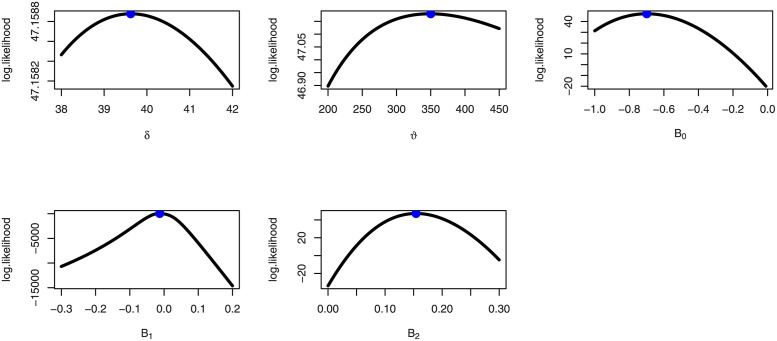
Profile likelihood function for food expenditure data.

Fig 3 provides the box plots for Mock Jurors data and food Expenditure data. Fig 4 discussed the profile likelihood function for food expenditure data which shows the likelihood estimators have maximum likelihood value.

### 7.3 Univariate modeling for Covid-19 data

Here, we will show how the UEL distribution may be used with Covid-19 data. Many other models are compared to the UEL distribution, including Kumaraswamy (Kom), Beta, Kumaraswamy Kumaraswamy (K-K) [38], Topp-Leone generalized exponential (TLGE) [39], Marshall-Olkin Kumaraswamy (MOK) [40], Topp-Leone Weibull Lomax (TLWL) [41], and type II power Topp-Leone inverse exponential (TIIPTLIE) [42]. The data contain 38 days of Covid-19 data from Saudi Arabia, from 22 July 2021 to 28 August 2021 (https://covid19.who.int). These results illustrate the drought mortality rate. The following data are listed in Table 7.

**Table 7:** COVID-19 data for Saudi Arabia.

0.2375 0.2962 0.2167 0.2752 0.2353 0.2347 0.1951 0.2140 0.2329 0.2711 0.2126 0.2314 0.1924
0.2113 0.2683 0.2487 0.2674 0.1716 0.2666 0.2091 0.2278 0.1706 0.2271 0.1890 0.2077
0.2452 0.1319 0.2259 0.1504 0.1879 0.1689 0.2063 0.2249 0.1686 0.1310 0.1497 0.1309 0.1495

The MLEs and their SEs for the investigated models are provided in Table 8.

**Table 8:** MLE for distinct models with SE.

		*δ*	λ	*ϑ*	*β*
UEL	Estimates	456.0536	0.0126	4708.5437	
	SE	56.7608	0.0014	6320.9528	
TIIPTLIE	Estimates	2476.431	0.5786	0.2592	
	SE	7533.562	0.0844	0.415	
TLGE	Estimates	0.3718	19.3227	129.5283	
	SE	0.2097	2.1837	89.6524	
TLWL	Estimates	0.4115	3.6183	1.433	7.1159
	SE	1.893	2.361	1.4707	31.3617
K-K	Estimates	1.8895	3.2134	21.9457	27.7825
	SE	7.5985	9.9317	2.9463	247.3502
MOK	Estimates	0.0028	5.3491	12.3332	
	SE	0.0009	0.6685	13.3336	
Kom	Estimates	3.2736	125.2235		
	SE	0.277	49.047		
Beta	Estimates	18.1391	68.2481		
	SE	4.1248	15.6777		

**Fig 5:**
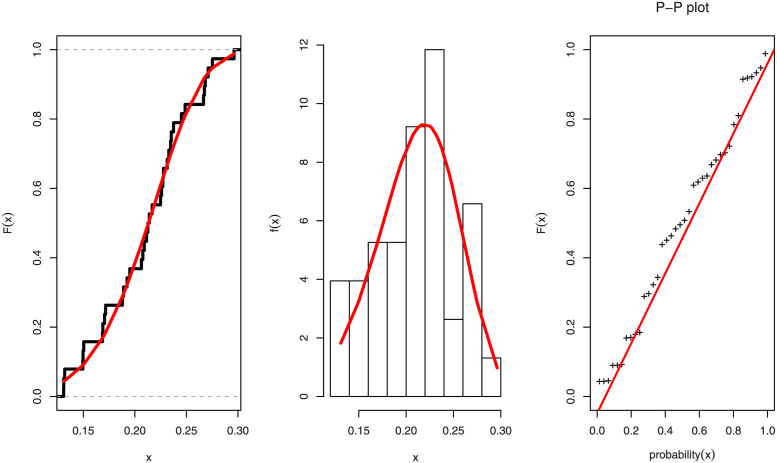
The empirical CDF, histogram, and P-P plot for the UEL distribution for Covid-19 data of Saudi Arabia.

For all fitted models using the Covid-19 data set, Table 9 shows the values of the AIC, BIC, CAIC, HQIC, CVMC, ADC, and KSD statistics, as well as their related P-values. Table 9 shows that the UEL distribution has the largest negative AIC, BIC, CAIC, HQIC, and P-value, as well as the smallest KSD, CVMC, and ADC values when compared to the other models used to fit the Covid-19. The empirical, histogram and PP-plot for the UEL distribution for Saudi Arabia’s Covid-19 data are shown in Fig 5.

**Table 9:** Various metrics for comparing various models.

	KSD	P-Value	-AIC	-BIC	-CAIC	-HQIC	CVMC	ADC
UEL	0.0787	0.9580	126.3390	121.4263	125.6332	124.5911	0.0358	0.2811
TIIPTLIE	0.1032	0.7752	125.6686	120.7558	124.9627	123.9207	0.0634	0.4156
TLGE	0.1475	0.3457	123.1948	118.2820	122.4889	121.4468	0.1115	0.6874
Kom	0.1509	0.3198	113.6135	110.3383	113.2707	112.4482	0.0520	0.3551
Beta	0.1185	0.6174	125.9104	120.6353	125.5676	123.7452	0.0788	0.5009
TLWL	0.0799	0.9526	124.3055	117.7552	123.0934	121.9750	0.0373	0.2861
K-K	0.0814	0.9452	124.3154	117.7650	123.1033	121.9848	0.0376	0.2875
MOK	0.1553	0.2877	113.9415	109.0287	113.2356	112.1935	0.1115	0.6912

## 8 Summary and conclusion

This study proposes the unit-exponentiated Lomax distribution, based on an appropriate transformation, which is useful for modeling data on the unit interval. Some mathematical characteristics of the UEL distribution are explored, such as moments, PWMs, IM, entropy measures, and stress-strength reliability. The maximum likelihood, Bayesian, and maximum product of spacing methods are employed for estimating the parameter of the suggested distribution. Results from simulations show that the criteria measurements of Bayesian estimates are preferred to comparable alternative estimates. It can be demonstrated that the UEL regression model is a reasonable alternative to unit-Weibull regression, beta regression, and the original linear regression models when using mock jurors and food spending data. Using Covid-19 data, the proposed model outperforms the beta, Kumaraswamy-Kumaraswamy, Topp-Leone generalized exponential, Marshall-Olkin Kumaraswamy, Topp-Leone Weibull Lomax, and type II power Topp-Leone inverse exponential across a variety of comparison criteria. In future research, we will discuss the application of UEL distribution based on these points as [43–51].
